# A comprehensive approach to simulating bone fractures through bone model fragmentation guided by fracture patterns

**DOI:** 10.1007/s11517-025-03428-5

**Published:** 2025-08-26

**Authors:** Gema Parra-Cabrera, Francisco Daniel Pérez-Cano, José Javier Reyes-Lagos, Juan José Jiménez-Delgado

**Affiliations:** 1 Computer Science Department, University of Jaén, Campus Las Lagunillas S/N, 23071 Jaén, Spain; 2 Department of Computer Languages and Systems, University of Granada, 18071 Granada, Spain; 3 Facultad de Medicina, Universidad Autónoma del Estado de México, Toluca, 50180 Mexico

**Keywords:** Clinical validation, Bone fracture simulation, Medical imaging, 3D models, Triangulation

## Abstract

**Abstract:**

Bone fractures are a common medical condition requiring accurate simulation for diagnosis and treatment planning. This study introduces a comprehensive method for simulating bone fractures using two-dimensional fracture patterns and real fractured bones applied to three-dimensional bone models. The approach begins with selecting and adjusting a fracture pattern, projecting it onto a 3D bone model and applying triangulation guided by quality metrics to simulate the cortical layer. Perturbation techniques add irregularities to the fracture surface, enhancing realism. Validation involved comparing simulated fragments with real fragments obtained from CT scans to ensure accuracy. Fracture patterns derived from real fragments were applied to non-fractured bone models to generate simulated fragments. A comparison of real and simulated fracture zones verified the minimal deviation in the results. Specifically, the distance between MMAR and MMAS scaled values varies between $-$0.36 and 1.44, confirming the accuracy of the simulation. The resulting models have diverse applications, such as accurate surgical planning, enhanced training, and medical simulation. These models also support personalized medicine by improving patient-specific surgical interventions. This advancement has the potential to significantly enhance fracture treatment strategies and elevate overall patient care.

**Graphical abstract:**

Pipeline for generating and validating 3D bone fracture patterns from CT models
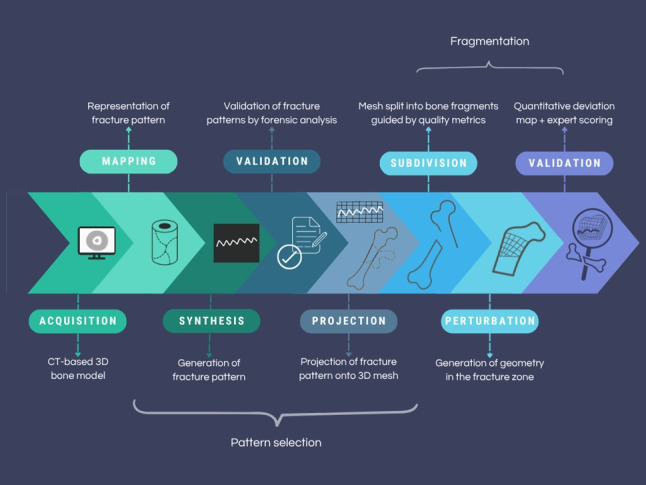

## Introduction

Accurately replicating bone fracture geometry is essential for orthopedic research, surgical planning, biomechanics studies, prosthetics development, and medical training [1–4]. Bone models are vital in visualizing skeletal structures, analyzing biomechanics, and supporting preoperative decision-making [5–7]. Advances in 3D modeling and printing technologies have enabled high-fidelity anatomical reconstructions with physical realism [8].

As emphasized by Moolenaar et al. [9], realistic fracture modeling is a key component in advancing computer-assisted preoperative planning. However, conventional fracture simulation methods, including manual modeling, finite element analysis (FEA), voxel-based, empirical-based, and physics-based approaches, have significant limitations. Although FEA using CT scans can replicate complex fracture geometries with high precision, as demonstrated by Inacio et al. [10], it relies heavily on patient-specific imaging and material parameterization [11–20]. Additionally, ex vivo mechanical testing provides valuable fracture parameters [1], but simulations based on them often fail to reflect clinical fracture morphology.

Avoiding the need for new CT imaging may reduce exposure to radiation, decrease acquisition time and cost, and simplify planning workflows. Projecting validated fracture patterns, either extracted from real cases or generated manually, onto intact bone models enables the generation of anatomically plausible fractures without individual imaging. This approach is especially useful in training, biomechanical modeling, and decision support, where the availability of diverse and realistic fracture patterns remains limited.

Various methods have been proposed to represent and compare fracture morphology, including point clouds and height maps [21], contour plots [22], and cylindrical coordinates [23]. Other studies have employed integrated models from cadaveric bones [24, 25], although validation remains challenging [26]. Alternative strategies—such as geometry-based, physics-based, or example-based simulation methods—enable fracture approximation without requiring full biomechanical modeling [27]. Fracture zone identification has also been approached using point-based selection and ICP algorithms [28–30], curvature analysis [31], and normal-based filtering [32]. These techniques allow the geometric extraction of fracture zones from 3D models with a focus on spatial accuracy. Recent advances also include applications in virtual reality for training [33, 34] and the use of machine learning for fracture prediction and classification, although these often require large datasets for training.

This work builds on previous contributions by simulating cortical bone fractures through the projection of validated fracture patterns onto intact bone models. These patterns may be manually designed [35], generated parametrically [36], or extracted from anonymized CT scans of real fractures and validated by forensic analysis [37]. The methodology accounts for both the outer and inner cortical layers, applies Delaunay triangulation and perturbation techniques [38] to reproduce surface irregularities, and performs geometric fragmentation of the model to generate separate bone fragments. Fracture classification follows AO/OTA standards [39], covering transverse, oblique, spiral, wedge, and comminuted fractures.

Compared to earlier approaches that only project fracture lines [38], this method performs actual fragmentation of the mesh, producing anatomically detailed and high-quality triangulations. Perturbation techniques enhance realism by simulating the roughness observed in real fracture surfaces, improving the clinical and visual plausibility of the simulated fractures.

Validation includes a quantitative comparison using height maps generated from both simulated and real fracture surfaces, where average height differences are calculated to assess deviation. Additionally, clinical experts in traumatology perform a qualitative evaluation of the anatomical plausibility, trajectory, and morphology of the simulated fractures. While this evaluation is exploratory, it supports the visual fidelity of the generated fractures.

Ensuring a high degree of clinical relevance in simulated fractures is essential for their applicability in medical practice. In this context, clinical accuracy refers to the ability of the simulation to replicate bone fractures realistically in terms of their anatomical location, morphological complexity, and propagation characteristics. This is assessed through the comparison of simulated fracture patterns with those extracted from CT scans of real clinical cases. Additionally, the method evaluates the geometric fidelity using 3D height maps and validates the fracture trajectory and surface roughness through expert clinical assessment.

The main objective of this study is to develop a reproducible and semi-automatic methodology for simulating realistic cortical bone fractures on 3D models without requiring new CT imaging. By balancing anatomical accuracy and modeling flexibility, this approach aims to support surgical planning, biomechanical simulation, and medical education.

This paper is structured as follows: the next section details the proposed simulation pipeline. Then, the Results and Discussion sections present the main outcomes, including validation against real CT-derived fractures and an analysis of potential applications. Finally, the Conclusions and future work section summarize the findings and outline future research directions.

## Methods

This methodology simulates realistic bone fractures by applying two-dimensional fracture patterns onto three-dimensional models of intact bones. The full process involves the following: (1) segmenting CT scans of both fractured and intact bones to isolate cortical structures, (2) reconstructing high-fidelity triangular meshes, (3) generating or extracting fracture patterns from fractured bone data, (4) projecting these patterns onto intact bone models, (5) fragmenting the bone mesh based on the projected pattern using triangulation and perturbation techniques, and (6) validating the results through both quantitative and expert-based visual assessment. Throughout the process, a clear distinction is maintained between fractured bone CTs (used for extracting real fracture data and for validation) and intact bone CTs, which serve as the base for applying and simulating the fractures.

To provide a structured overview of the methodology, Algorithm 1 summarizes the full simulation pipeline used to generate and validate 3D fractured bone models.

**Algorithm 1:**
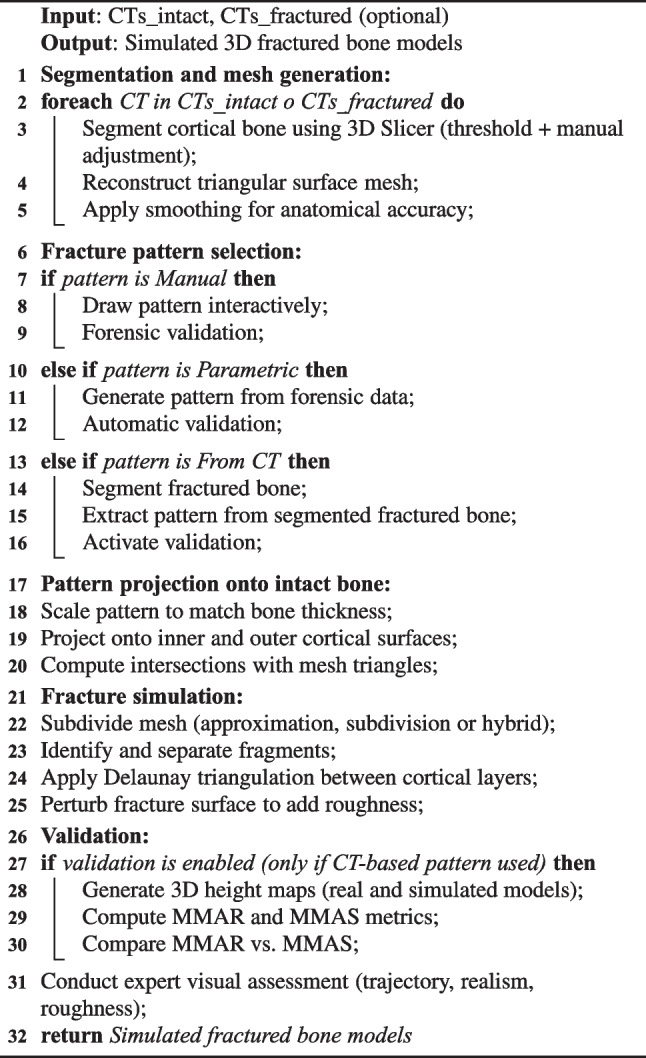
Summary of the bone fracture simulation process.

### Obtaining and preparation of bone models

The simulation process employs 3D triangular mesh models derived from CT scans of both intact and fractured bones (including femur, fibula, humerus, and radius). Fractured bone CTs were used exclusively for pattern extraction and validation, while intact bone CTs served as the base models for applying fracture simulations. Segmentation was performed using 3D Slicer [40]. Manual refinement ensured clean anatomical boundaries and consistent surface quality across all cases. The resulting segmentations were reconstructed into high-fidelity triangular meshes suitable for subsequent simulation steps.

Triangular meshes balance detail and computational efficiency [41], making them well suited for applications in surgical planning, prosthetic design, and virtual reality applications. These meshes are typically generated from CT-derived point clouds or voxel data using surface reconstruction algorithms, providing continuous and manipulable representations of bone geometry. Compared to voxel-based models, triangular meshes have been shown to better represent superficial bone features and deformities [42].

To enhance surface accuracy, advanced segmentation and meshing techniques were applied [4, 43]. Smoothing algorithms [44] were used to reduce aliasing and ensure surface continuity by eliminating abrupt curvature transitions. This refinement improves anatomical realism and ensures the structural quality required for high-fidelity fracture simulation.

### Selection of fracture patterns

Fracture patterns define the geometry and propagation of bone fractures and are central to the simulation process. In this work, patterns are generated or extracted in two dimensions and then projected onto 3D bone models to simulate realistic fractures. This section details how patterns are represented, created, validated, and applied.

#### Pattern representation

Fracture patterns serve as 2D geometric descriptors of fractured regions, derived from medical images or forensic analysis. Common representations include spherical coordinates, 2D textures, and 3D height maps [36, 45]. In this study, patterns are flattened onto a 2D plane to facilitate projection onto bone models, as proposed by Cohen et al. [23]. The approach incorporates detailed segment information to preserve the morphology of real fractures and is informed by forensic criteria [23, 37, 46] to ensure clinical relevance.

**Fig. 1:**
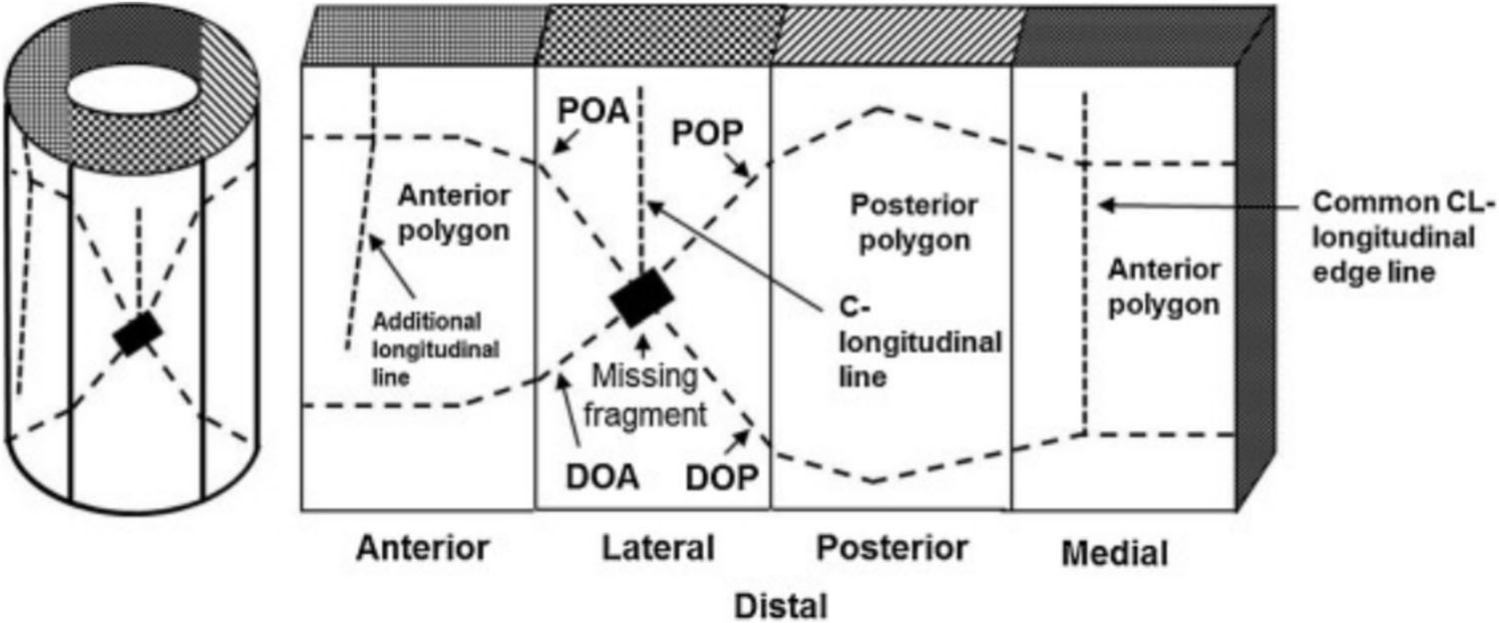
Representation of a fracture on a cylindrical model (left) and its two-dimensional projection (right) according to the method proposed by Cohen et al. [23]. This process involves transforming the 3D fracture data into a 2D format for easier analysis and application in simulations. Image extracted from Cohen et al. [23]

#### Pattern generation

The generation of fracture patterns began with creating 2D patterns (Fig. 1) using specialized tools, including software and algorithms. These patterns aimed to replicate fractures in the diaphysis of long bones, based on classifications like AO/OTA [39]. Three approaches were used: interactive drawing of patterns, automatic parametric generation, and extraction from real fractured bone models via CT scans. The first two approaches required validation of the generated patterns.

- **Interactive pattern generation **using a graphical editor [35], allowing users to manually create fracture lines and define key attributes such as location, orientation, shape, and the presence of detached fragments.
- **Automated parametric generation**, following the method proposed by Jiménez-Delgado et al. [36], produces two-dimensional fracture patterns (Fig. 1) based on predefined forensic parameters [37, 46]. The system uses clinical data to define geometric features for each fracture type [39], enabling consistent and realistic simulations without manual intervention. Pattern accuracy is ensured through automated validation tools [35, 37], which verify coherence with forensic criteria and were developed to streamline and support the generation, analysis, and validation of fracture patterns in a reproducible way.
- **Pattern extraction from CT scans of fractured bones**, using the algorithm proposed by Luque et al. [47]. This method identifies fracture zones by combining geometric discretization techniques—such as oriented bounding boxes (OBB), grid structures, curvature analysis and surface normals—with seed-based filtering. The algorithm classifies points into outer and inner cortical layer and projects them into 2D space, generating anatomically realistic fracture patterns that capture both propagation and morphology.

#### Pattern validation by forensic analysis

Fracture patterns were validated using forensic analysis tools that compare geometric and morphological features with documented clinical cases [23, 37, 46]. The automated validation system applies predefined criteria based on forensic studies [36], valuating each pattern according to three key aspects: the number and classification of fracture lines, their lengths, and the presence and distribution of fragments along the bone axis.

These criteria are derived from statistical analyses of real fractures and ensure that generated patterns are consistent with clinically observed morphologies. Patterns meeting all thresholds are considered valid for simulation. Full details of the validation process can be found in Jiménez-Delgado et al. [36].

#### Pattern projection

Validated 2D patterns were projected onto 3D bone models using a method described in prior work [38]. The pattern was aligned and scaled based on the target bone’s thickness and curvature. A cylindrical approximation of the bone surface was used to unwrap the mesh, apply the pattern, and re-map it to 3D space. Intersections between the fracture line and mesh triangles defined the cutting points used in the fragmentation stage. Projection was carried out independently for both the inner and outer cortical layers, enabling dual-surface fracture simulation.

**Fig. 2:**
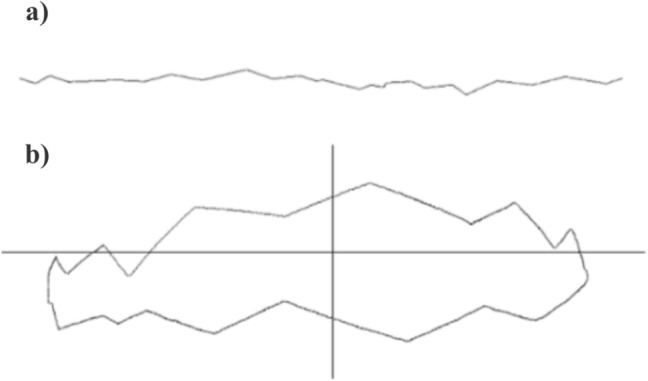
Schematic of the projection method used to apply 2D fracture patterns onto 3D bone models. **a** Original pattern (*P*). **b** Transformed pattern ($P'$)

The projection process began by selecting a suitable 2D fracture pattern *P* (Fig. 2a) and preparing the 3D bone model *M*, represented as a triangular mesh. The application height on the bone was then defined, and the pattern was scaled accordingly in both transverse and longitudinal axes to match the local bone thickness. A transformation *T* was applied to align and adapt the pattern to the model, resulting in the adjusted pattern $T(P) = P'$, where $P'$ is the pattern adjusted to model *M* (Fig. 2b), ready for projection onto the mesh surface.

**Fig. 3:**
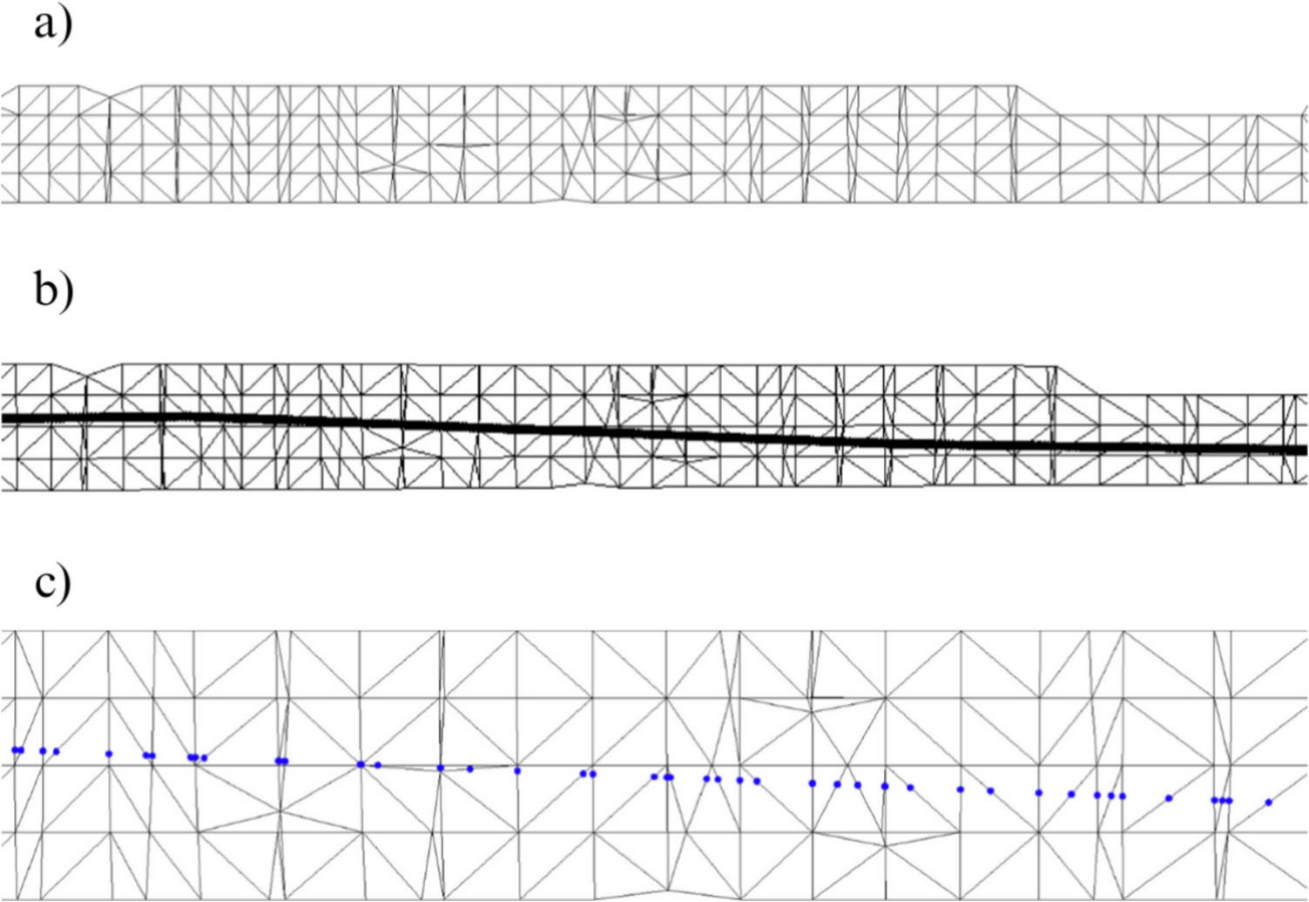
Different representations of fracture zone. **a** 2D fracture zone. **b** 2D fracture zone with the fracture pattern superimposed. **c** 2D fracture zone with the shear points on its triangles

**Fig. 4:**
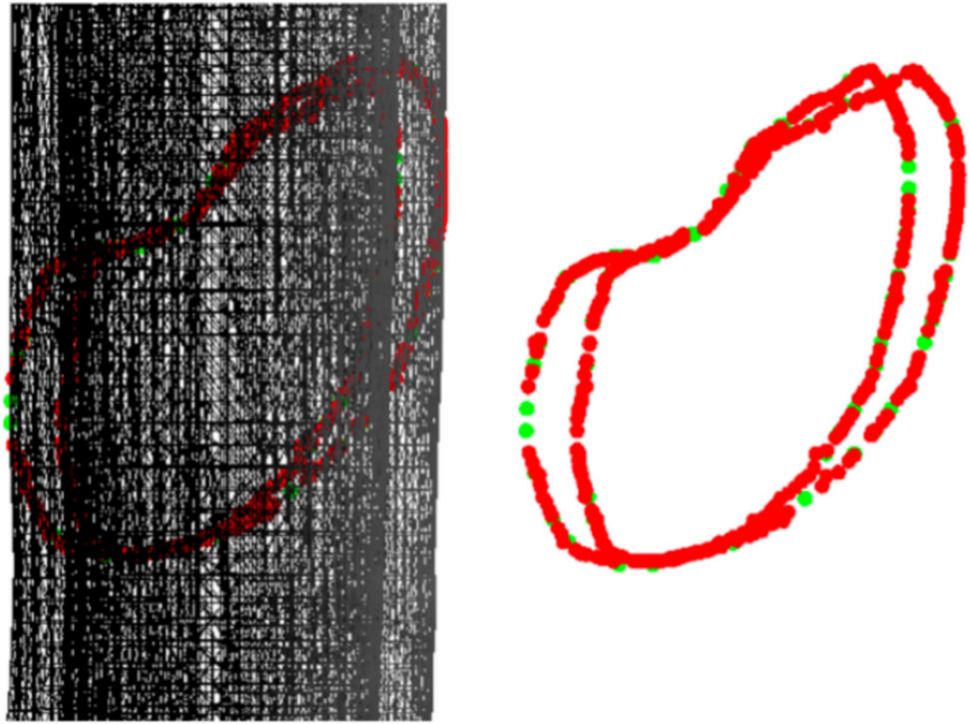
Points belonging to the bone fracture in the inside and outside layers of the model. Points colored in green belong to the triangles whose cut points follow approximation strategy and those colored in red represent the triangles on which a subdivision is made

To support the projection process, the target region of the bone was enclosed within a cylindrical structure. Given the natural irregularities of the bone surface, the cylinder radius was defined based on the bone’s thickness at the intended fracture height. The triangle mesh was then adjusted to fit the cylinder, avoiding triangle overlap and transformed into 2D for calculations (Fig. 3a). The fracture pattern was applied and its width adjusted to match the bone’s thickness, corresponding to the width of selected triangles (Fig. 3b).

Intersections between the fracture pattern and the bone mesh were then calculated to identify the precise cutting points along the surface (Fig. 3c). These points defined where the fracture lines intersected the mesh triangles, enabling their direct integration into the bone geometry rather than a superficial overlay. As illustrated in Fig. 4 (left), this process established a topological correspondence between the projected fracture pattern and the 3D mesh, ensuring an anatomically coherent fracture representation.

### Fragmentation of bone models and fracture zone generation

Following the projection of the fracture pattern, the bone model was subdivided to determine bone fragments. Using triangulation, adjacent triangles were connected coherently to realistically depict the fracture across cortical tissue layers. Perturbation techniques introduced surface irregularities, mimicking natural variations in real fractures to enhance model realism.

#### Subdivision of the bone model guided by quality metrics

**Fig. 5:**
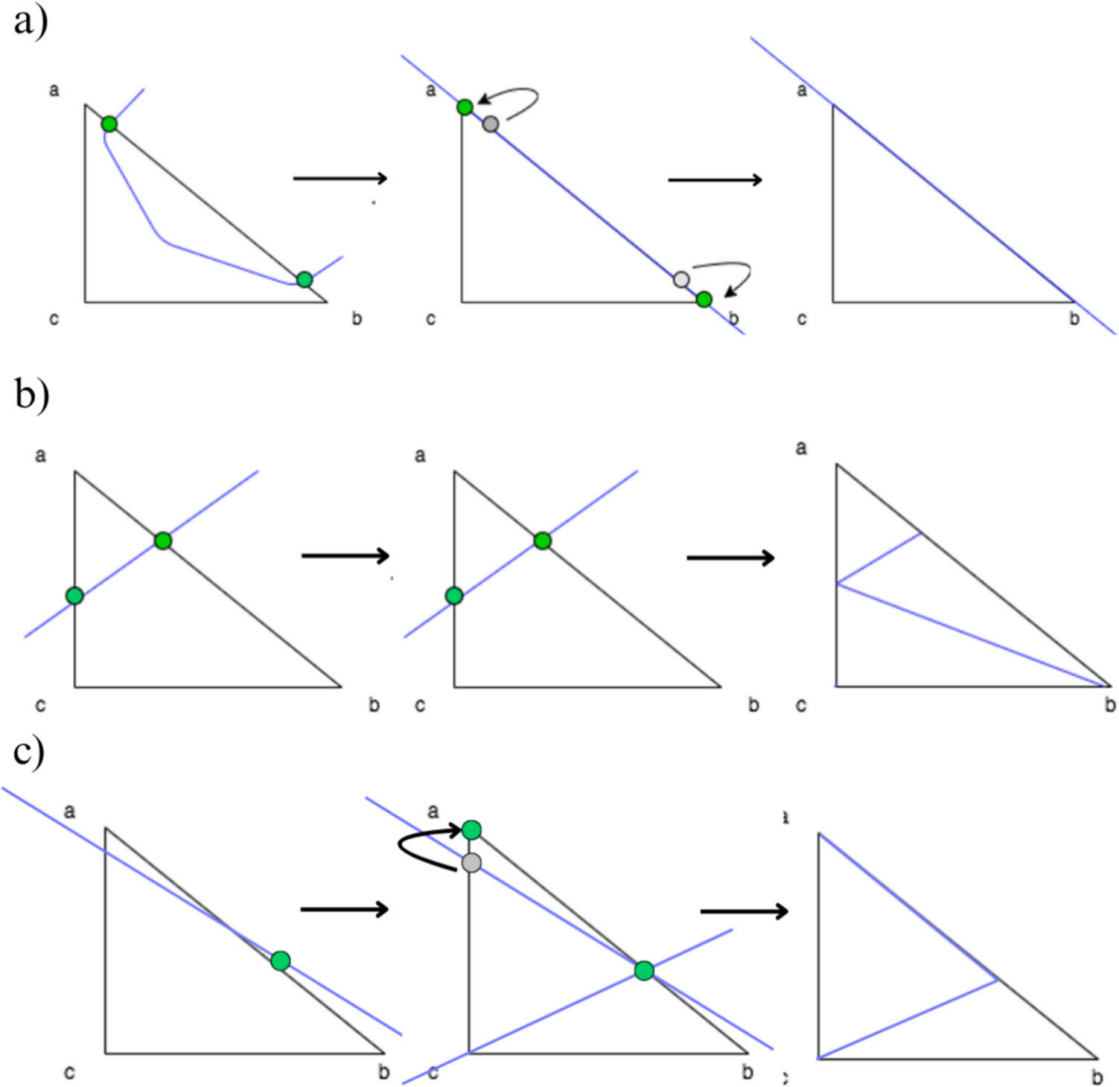
Comparison of approximation (**a**), subdivision (**b**), and hybrid (**c**) strategies for cutting points in the bone model mesh

Subdividing the bone model into fragments followed the fracture pattern projection, involving determining cutting points along the triangles of the bone mesh model. Methods include approximation, subdivision, and hybrid approaches [48]. In the approximation method (Fig. 5a), cutting points were moved to the nearest vertex, maintaining mesh topology. Subdivision method (Fig. 5b) retained original cutting point positions, forming new triangles from existing vertices. The hybrid approach (Fig. 5c) combined these methods, optimizing mesh quality while ensuring fracture precision with an adjustable approximation threshold.

The subdivision of triangles generated from cut points and vertices is addressed using quality criteria to optimize the geometric representation of fragments. Based on the principles established by Knupp et al. [49], a comprehensive analysis [48] was conducted to identify the most suitable criterion for ensuring the average mesh quality. As a result, the metric based on the ratio of inradius (*r*) to circumradius (*R*) was found to be the most effective for this purpose.

This metric evaluates the arrangement of triangle points [48], ensuring the formation of high-quality triangles with ideal quality values in the range [1, 3], where values approaching 1 correspond to equilateral triangles (Table 1). The use of this approach, combined with an approximation threshold of 10%, proved effective in ensuring an accurate representation of fracture geometry in bone meshes.

**Table 1:** Quality ranges for the ratio between circumradius and inradius of a triangle

Triangle radius ratio	
Dimension	1
Acceptable range	[1, 1.3]
Normal range	[1, DBL_MAX]
Full range	[1, DBL_MAX]
*q* for an equilateral triangle	1

**Fig. 6:**
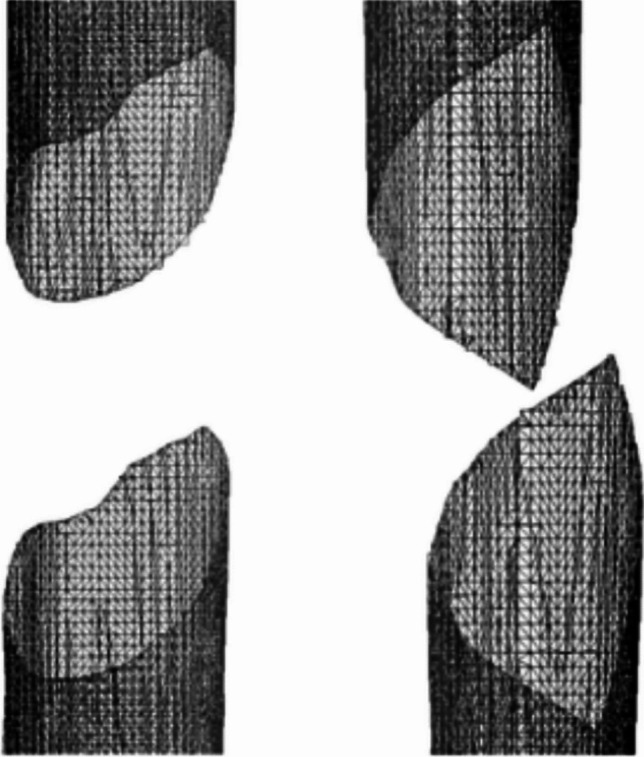
Bone model with the fragments identified. Spiral (left) and oblique (right)

The next step involved identifying fracture points in both the outer and inner layers of cortical tissue. These points result from intersecting the projected fracture pattern with the bone model, guiding the segmentation of the model into distinct bone fragments. Once the fracture zone was defined and the corresponding bone geometry established, each fragment and part of the cortical tissue, inside and outside layers, were identified for subdivision at the fracture site (Fig. 4). Detection and identification of different fragments proceed from a seed point, iterating through neighboring triangles until reaching those designated as part of the fracture line (Fig. 6).

#### Generation of geometry in the fracture zone

**Fig. 7:**
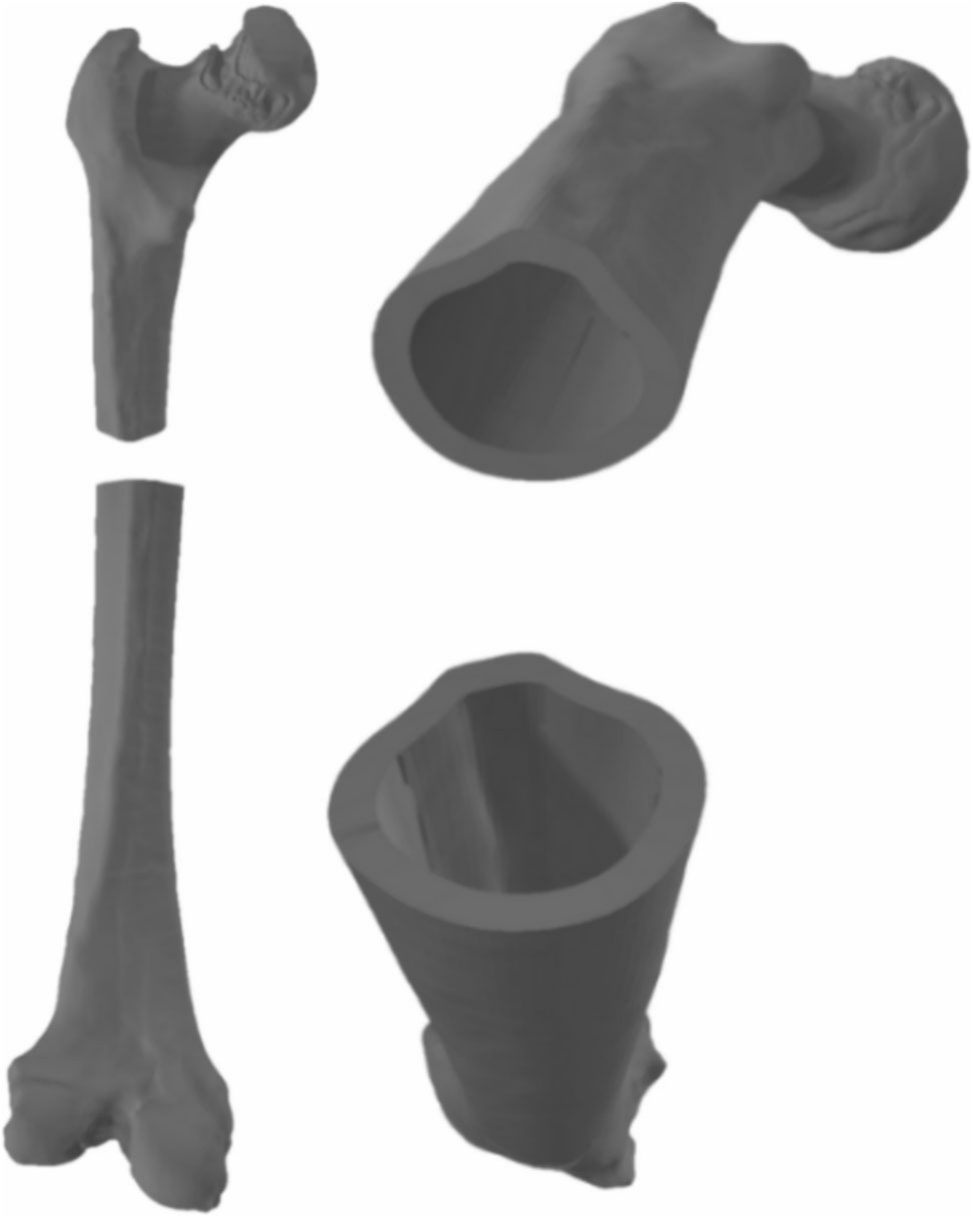
Transverse fracture applied on femur with generation of geometry on the fracture surface by Delaunay

The process connects the inner and outer cortical surfaces at the fracture zone using Delaunay triangulation. This method ensures a coherent and continuous connection between triangles, while applying specific constraints to avoid meshing regions that should remain hollow—such as the central medullary canal where trabecular bone is located. Figure 7 illustrates the closure of a transverse fracture using Delaunay triangulation applied to the cortical surface.

**Fig. 8:**
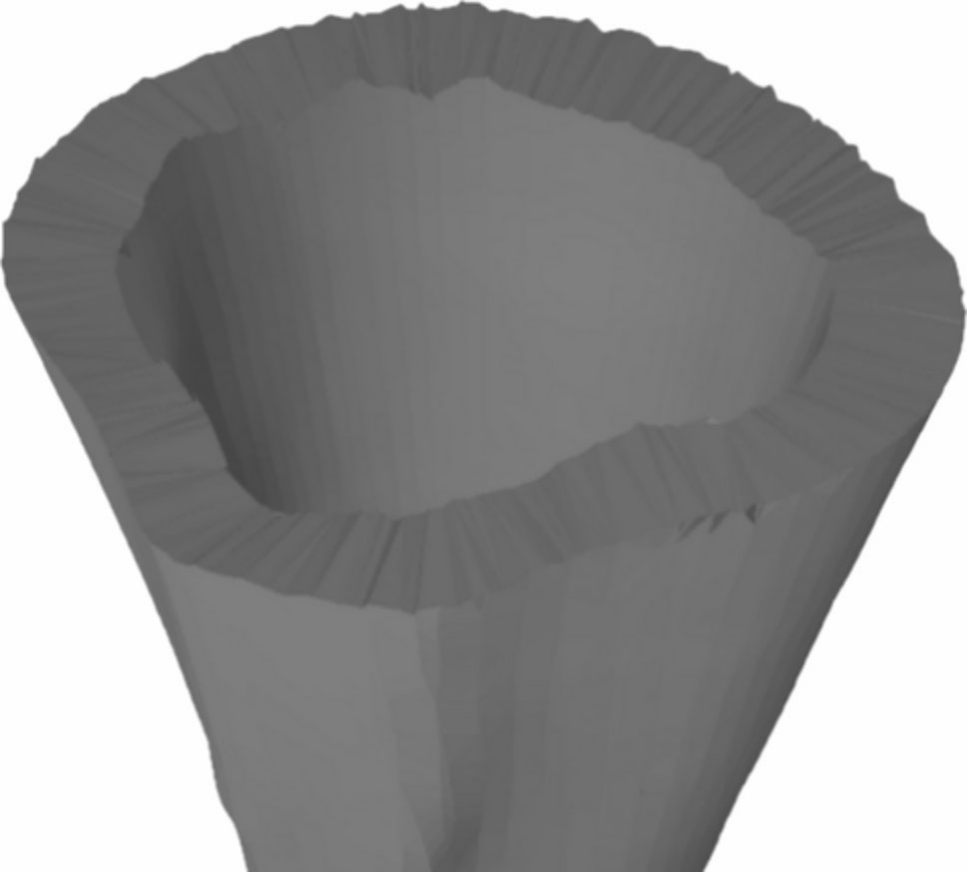
Fractured bone model following a transverse fracture pattern with a hybrid approach and the proposed geometry generation method without auxiliary points in the fracture zone

**Fig. 9:**
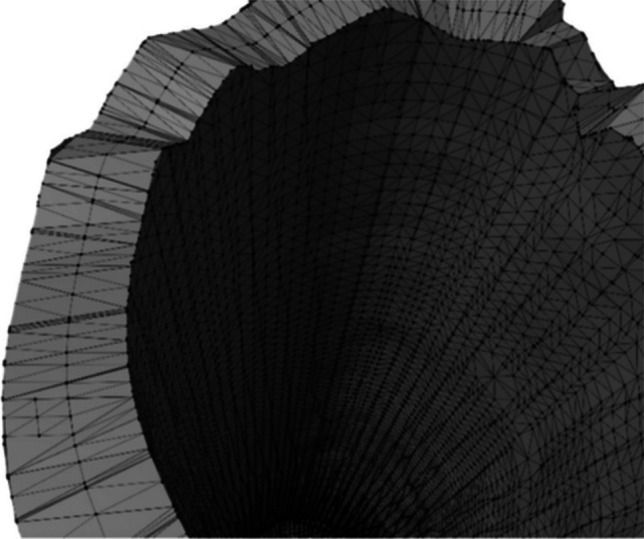
Initial triangulation of the fracture zone with auxiliary points. These points generate a perturbation effect, simulating the roughness observed in actual fractures

**Fig. 10:**
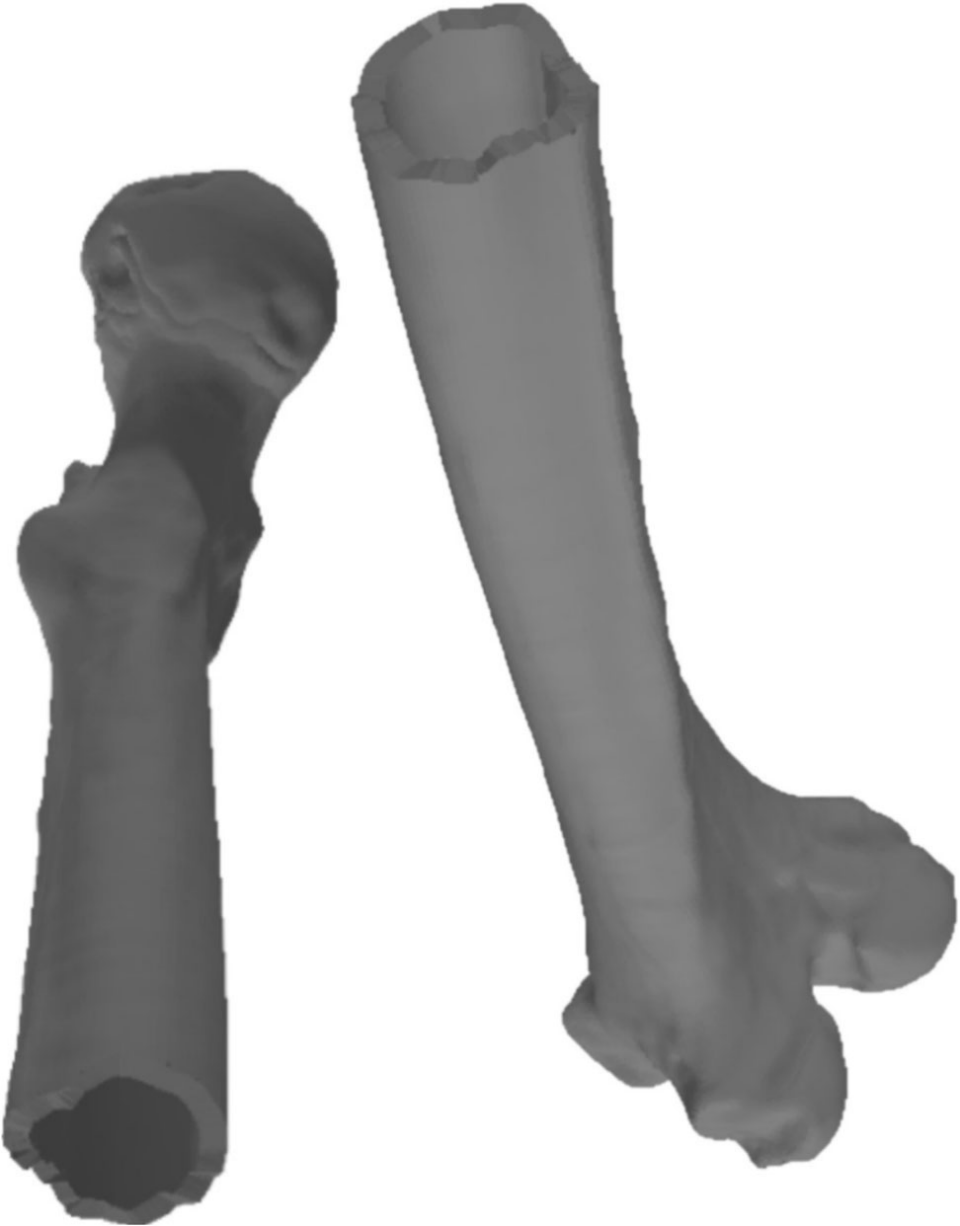
Fracture zone after applying perturbation. This process follows the triangulation and generation of new points in the fracture zone

To enhance realism in the fracture zone, two perturbation techniques were implemented. In the first method (Fig. 8), perturbations are applied directly to the vertices in the fracture zone before triangulation. Each vertex is displaced along its normal vector using a random value within a user-defined threshold, which controls the maximum magnitude of displacement. This threshold typically ranges from 0.1 to 1.0 mm, and these values have been adjusted experimentally to achieve a more realistic appearance of the fracture. As a result, the resulting fragments already incorporate surface irregularities at the time of triangulation. The second method (Fig. 9), unlike the previous one, applies perturbation after the initial triangulation of the fracture surface. In this case, an auxiliary point is added at the center of each triangle and displaced along the triangle’s normal vector (Fig. 10), again using random values uniformly distributed within the same 0.1–1.0 mm range. This approach introduces controlled surface roughness and mimics the non-uniformity typical of real bone fractures, enhancing both visual and anatomical realism.

### Model validation using fractures from CT scan

To evaluate the accuracy of the simulated fractures, a validation process was conducted by comparing the results against real fractured bone models derived from anonymized CT scans. These real models served as references for generating and applying fracture patterns to corresponding intact bone models. The goal was to assess how closely the simulation reproduces the morphology and trajectory of actual fractures.

**Fig. 11:**
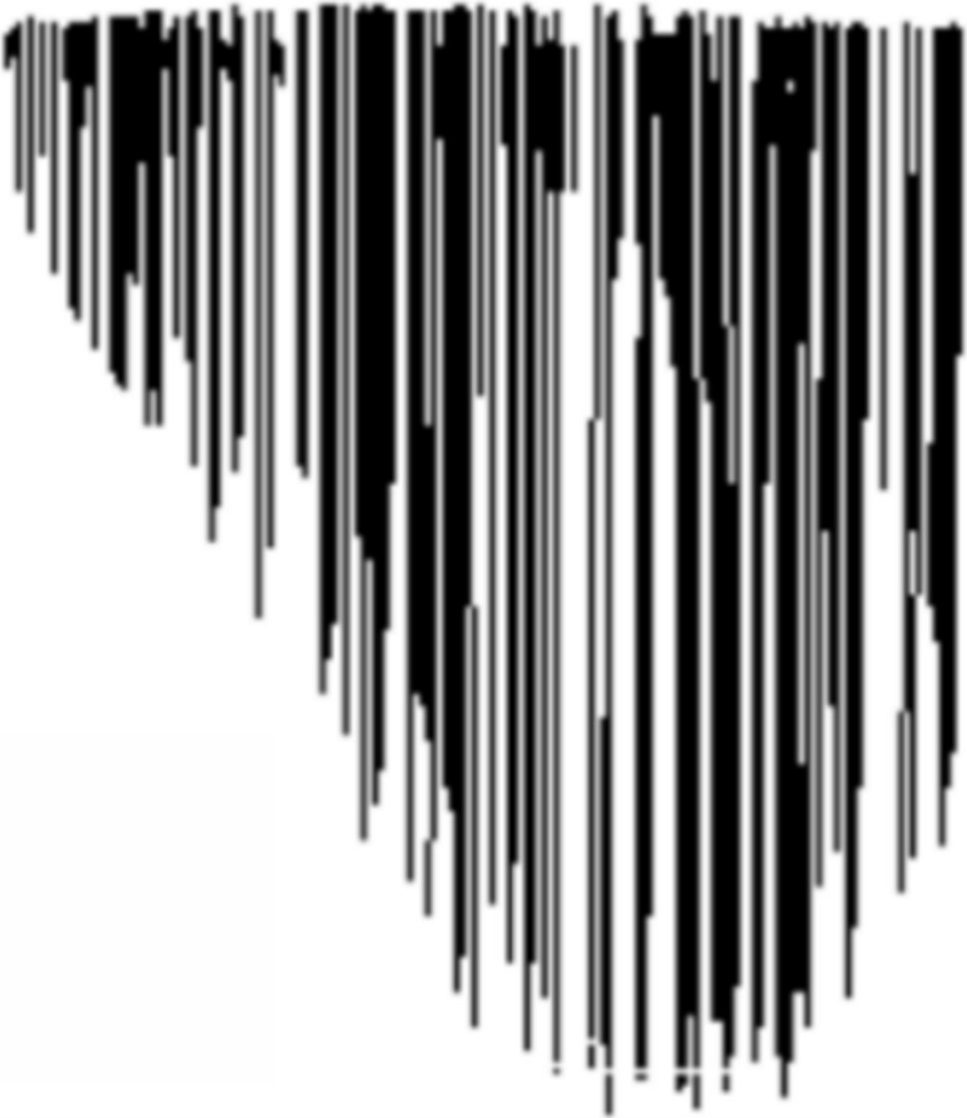
Representation of a fracture zone derived from a real fibula fracture using a height map. This map provides a three-dimensional visualization of the fracture, showing the depth and contours of the fracture lines

**Fig. 12:**
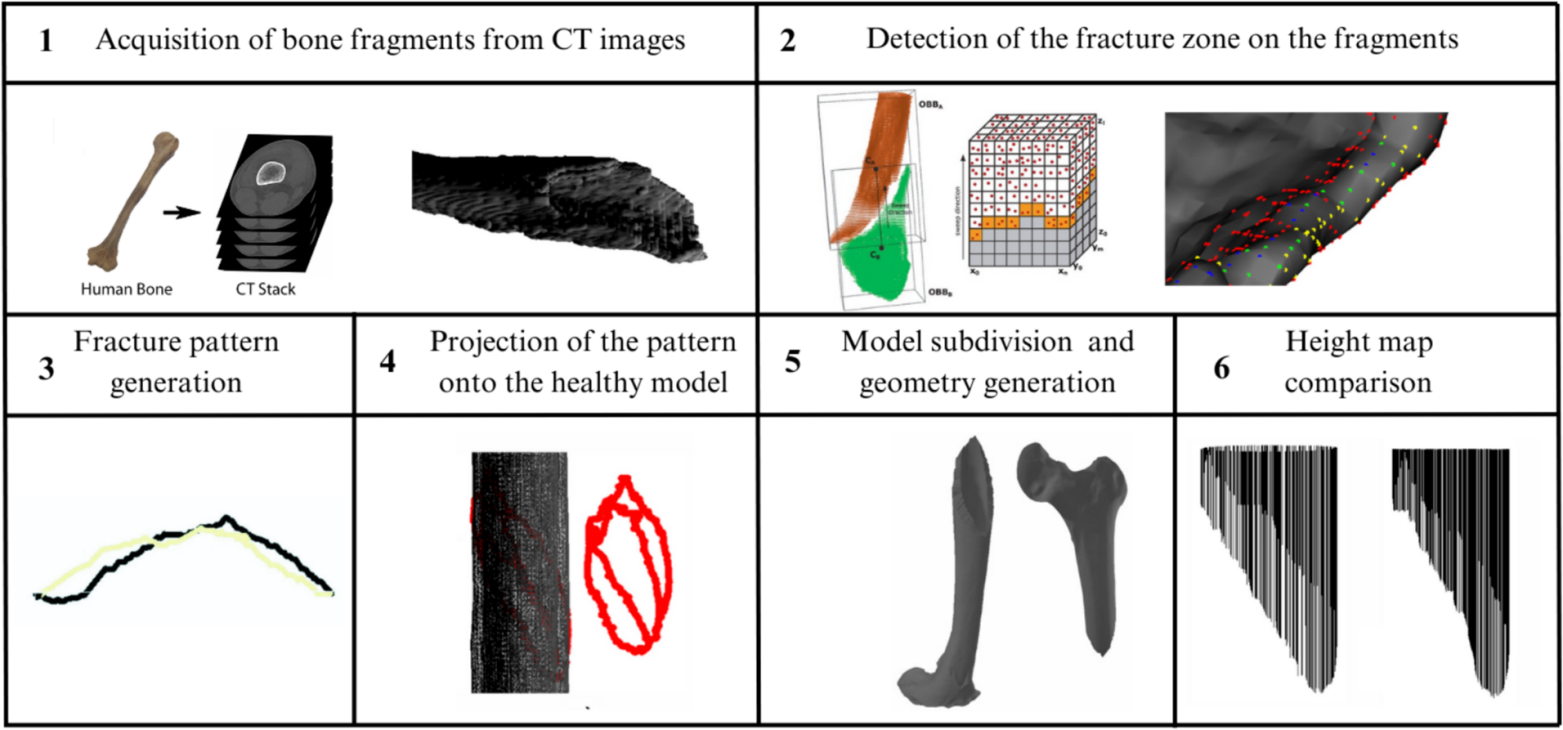
Overview of the fracturing process. This summarizes the steps from obtaining real fractures to applying patterns on 3D models and generating height maps for validation

The validation pipeline involved six sequential steps, illustrated in Fig. 12:

1. **Acquisition of fractured bone models:** 3D models of fractured bones were obtained through segmentation and reconstruction of CT images, generating volumetric and surface mesh representations.
2. **Detection of the fracture zone on the fragments:** Using the algorithm from Luque et al. [47], fracture zones were detected through a combination of oriented bounding box (OBB) discretization and a grid. The grid was traversed parallel to the sweep vector, marking candidate points in each column and filtering for points belonging to the outer or inner cortical layers, thereby identifying fracture points for both layers.
3. **Fracture pattern generation:** Points along the fracture edges were extracted and converted from 3D to 2D space, generating fracture patterns for both the external and internal layers, accounting for changes during fracture propagation.
4. **Projection of the pattern onto intact models:** These patterns were projected onto intact bone models using the method described in [38], aligning them anatomically and adjusting their scale to match the target bone’s geometry.
5. **Model subdivision and geometry generation:** The subdivision of the bone model used triangle subdivision techniques based on quality metrics [49]. This facilitated the identification of points belonging to the fracture line for each fragment. The cortical tissue layers were joined in the simulated fractured model through Delaunay triangulation and perturbation techniques.
6. **Height map comparison:** 3D height maps were generated based on the fracture zones of both simulated and real models. A reference plane was positioned above the fracture zone, from which perpendicular distances were measured to each surface point. These values formed a height profile used for quantitative and qualitative comparison (Fig. 11).

For validation, height maps of both the real and simulated bone models were compared. Specific features analyzed included the overall shape of the fracture surface, the roughness profile and the similarity of the fracture trajectory. Quantitative comparison was performed using the average height differences between corresponding points of the real and simulated fracture surfaces, measured along lines projected from a common reference plane. This metric, referred to as “height map distance” (MMAR for real models and MMAS for simulated ones), captures surface deviation while preserving the spatial distribution of the fracture morphology.

The validation process involved a multi-step comparison between simulated fractures and real fractured bone models obtained from anonymized CT scans. Fracture patterns extracted from real cases were applied to intact bone models, and both the real and simulated fractures were evaluated using height maps generated from the fracture zones. These maps enabled both quantitative and qualitative assessment of surface similarity.

Quantitative validation focused on comparing features such as the overall shape of the fracture surface, surface roughness, and fracture trajectory. For each case, a height map was constructed by projecting distances from a reference plane onto the fracture surface. The average absolute difference in height values between corresponding points on the real and simulated models was computed—referred to as the “height map distance.” This metric, expressed as MMAR (for real fractures) and MMAS (for simulated fractures), quantifies surface deviation while preserving spatial structure. The results are presented in Table 4.

Qualitative validation was independently conducted by a group of clinical experts. The experts visually assessed the simulated fractures based on three key criteria: (1) visual plausibility, (2) anatomical coherence, and (3) similarity to clinical cases. Evaluations were performed individually using a 3-point Likert scale (1 = low, 2 = moderate, 3 = high), drawing on the clinicians’ experience in fracture interpretation (Table 2).

**Table 2:** Expert scoring of simulated fractures using a 3-point Likert scale across three qualitative dimensions

Case ID	Expert	Anatomical coherence	Visual plausibility	Similarity to clinical cases	Global score
#01	A	3	3	3	2.92
	B	3	2	3	
	C	3	3	3	
	D	3	3	2	
#02	A	2	3	3	2.42
	B	2	3	2	
	C	2	2	3	
	D	3	2	2	
#03	A	2	2	3	2.33
	B	2	3	2	
	C	2	2	2	
	D	3	2	3	

**Scale:** 1 = low realism, 2 = moderate realism, 3 = high realism. The average score per case reflects the mean across all dimensions and experts

This structured scoring framework provided a consistent basis for discussion, leading to a consensus evaluation that determined the overall fidelity of each model. This validation process was carried out separately from the quantitative height map analysis and served as a complementary assessment of simulation realism.

Although this stage relied on consensus-based qualitative evaluation, future studies could incorporate formal inter-rater reliability analysis to reinforce the methodological robustness. Multiple experts could independently evaluate a representative sample of simulated fractures using the same criteria, with agreement levels quantified using statistical measures such as Fleiss’ kappa, which is appropriate for more than two raters.

The proposed simulation pipeline is semi-automatic and runs efficiently on standard hardware. Once a fracture pattern is selected, adapting it to a 3D bone model and generating the fractured output takes 0.20–0.78 s per case, depending on model complexity. Simulations were performed on a 2017 MacBook Pro (2.9 GHz Intel Core i7, 16 GB RAM) without GPU acceleration, confirming the method’s low computational demands.

The framework was developed in C++ and integrates several specialized libraries to ensure both computational performance and geometric accuracy. Among the most relevant, OpenMesh was used for mesh representation and manipulation throughout the pipeline, while OpenGL enabled interactive visualization of bone models and fracture patterns. Additionally, 3D Slicer served as the tool for segmenting DICOM-based CT data and reconstructing high-fidelity surface meshes from both intact and fractured bones.

Core components of the pipeline, including pattern projection, mesh subdivision, and fracture surface perturbation, were implemented using internally developed algorithms, designed for reproducibility and flexibility. The use of efficient data structures and optimized geometry processing routines allows the framework to handle anatomically complex fracture scenarios while preserving structural coherence and visual realism.

## Results

To demonstrate the effectiveness of the described fracture simulation process, fractured models have been generated as illustrative examples. These models serve as visual and practical representations intended to verify the ability to accurately summarize the characteristics of bone fractures in two-dimensional patterns and faithfully reproduce these characteristics in three-dimensional bone models.

This section presents the results of the fracture generation process on bone models using fracture patterns. It includes a subset of fractured models based on various patterns, fragments obtained from CT images of real fractures, and the validation results of the method, including expert visual validation, as described in the Methods section.

### Bone fracture simulation

Validation began with real fractured bone models (Figs. 12 (1) and 13), including a fibula and humerus, represented as triangle meshes. Obtaining these models from CT images is challenging, as they provide detailed bone geometry, density, and internal tissue arrangement. The CT scans used to generate the real fractured bone models were obtained from completely anonymized clinical cases provided by the University Hospital of Jaén, Spain. The process involves segmenting and processing CT images to capture bone shape, structure, and fracture details. These models serve as references for generating 2D patterns to apply over intact 3D bone models.

These fractures were applied to two intact bones (simulated models), the femur and the humerus, obtaining three viable combinations between the real model and the simulated model. Despite the variability in the source of the fracture patterns, their applicability to similar bones, such as the long bones of the diaphysis, is justified by the fracture pattern’s adaptability to the dimensions and peculiarities of the bone model to be fractured. Table 3 shows the number of vertices and triangles for each original and simulated model. These models were validated by comparing them with the real fractures through the analysis of height maps, as described in Section 3.2. This allowed a quantitative assessment of surface similarity between simulated and real fracture zones.

**Fig. 13:**
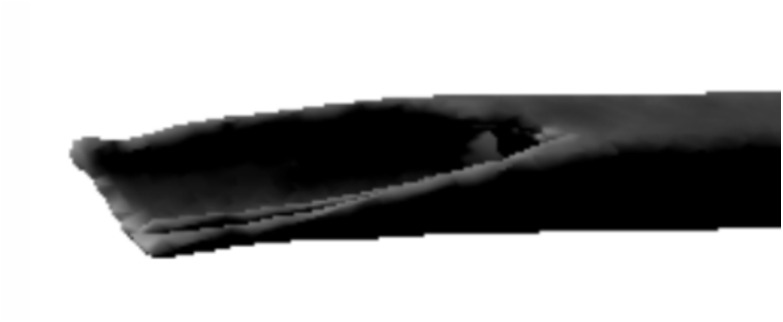
Models of real fractured bone (humerus)

**Table 3:** Number of vertices and triangles by original model (intact or fractured) and by simulated model (pattern obtained from a real fractured bone and applied on a different model)

Model	Intact femur	Intact humerus	Fractured fibula	Fractured humerus	Fibula fracture pattern applied on femur	Fibula fracture pattern applied on humerus	Humerus fracture pattern applied on femur
Vertex	970,104	100,158	173,376	55,264	570,351	56,511	580,998
Triangles	335,368	33,386	57,792	110,528	190,117	18,837	193,666

An additional model, a radius, was used in the tests, but due to the dimensions of the real models in relation to the size of this type of bone, the application of this type of fracture was not feasible. When a fracture obtained from a bone of larger dimensions is applied to a bone with a considerably smaller radius, adapting the fracture presents difficulties due to the difference in scale. An adjustment must be made to maintain the aspect ratio in the pattern, ensuring it makes sense for the bone to which it is applied.

**Fig. 14:**
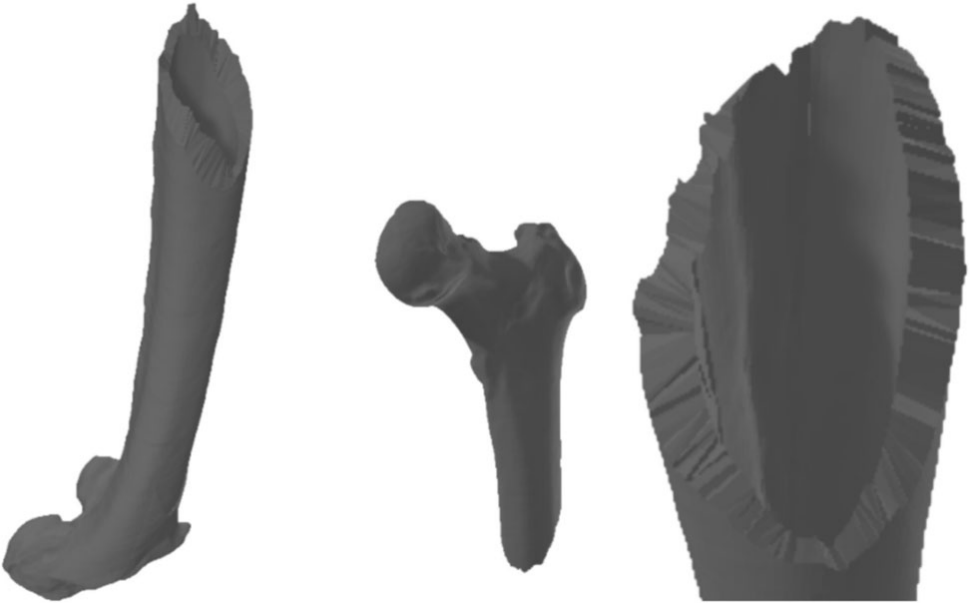
Simulation of a fractured femur using a fracture pattern originally extracted from a real fibula. The left and central images show the femur model after pattern projection and fracture simulation. The right image presents a detailed view of the fracture zone, emphasizing surface morphology and mesh structure resulting from Delaunay triangulation and perturbation

**Fig. 15:**
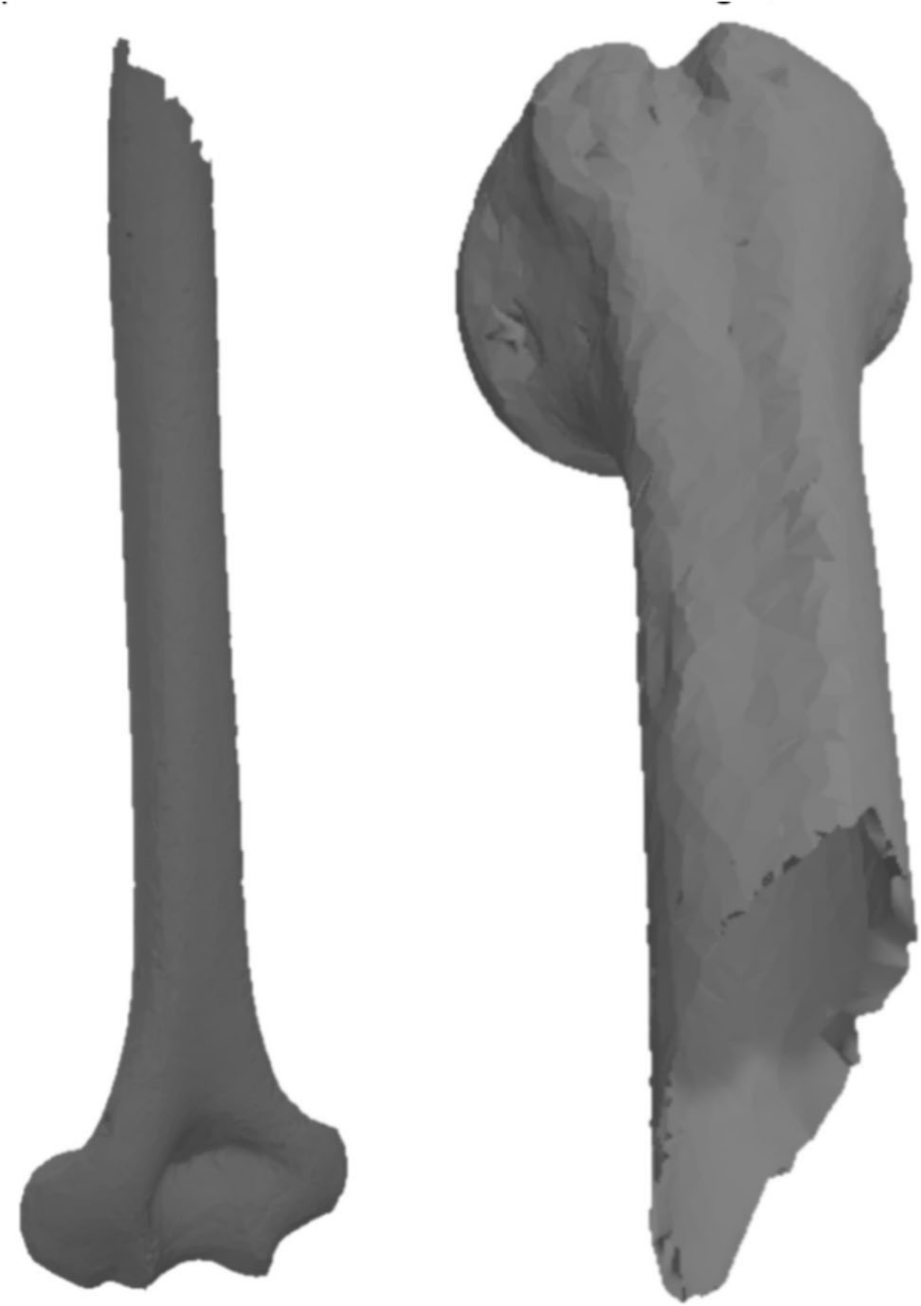
This figure shows a simulated humerus fracture generated by applying a fracture pattern extracted from a real fibula. The result illustrates the adaptability of the method to different anatomical models while preserving the characteristic morphology of the original fracture pattern

In one of the simulated cases, an oblique fracture of a fractured fibula was obtained and applied to a femur (Fig. 14). Similarly, the fracture of a real fractured humerus was obtained and applied to a femur. Finally, a fracture pattern of a fractured fibula with a large number of irregularities was obtained and applied to a humerus to simulate the fracture (Fig. 15).

**Fig. 16:**
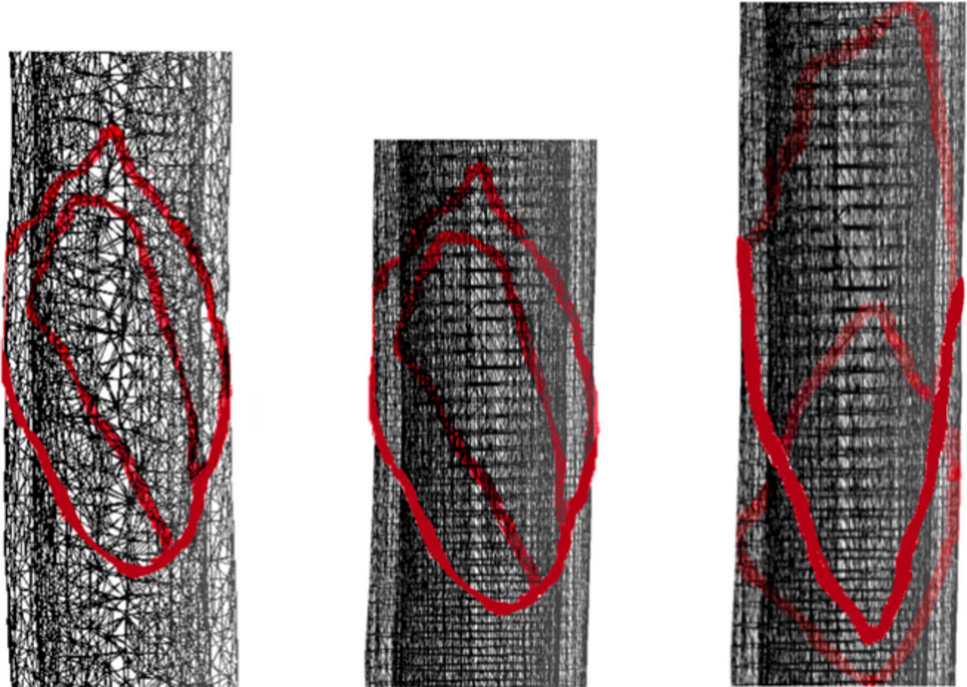
Projection of 3D cut points onto the cortical layer of bone models, illustrating the fracture pattern applied to both the outer and inner cortical layers. **a** Fracture pattern extracted from a fibula and applied to a humerus. **b** Same fibula-derived pattern applied to a femur. **c** Pattern extracted from a fractured femur and applied to a femur

**Fig. 17:**
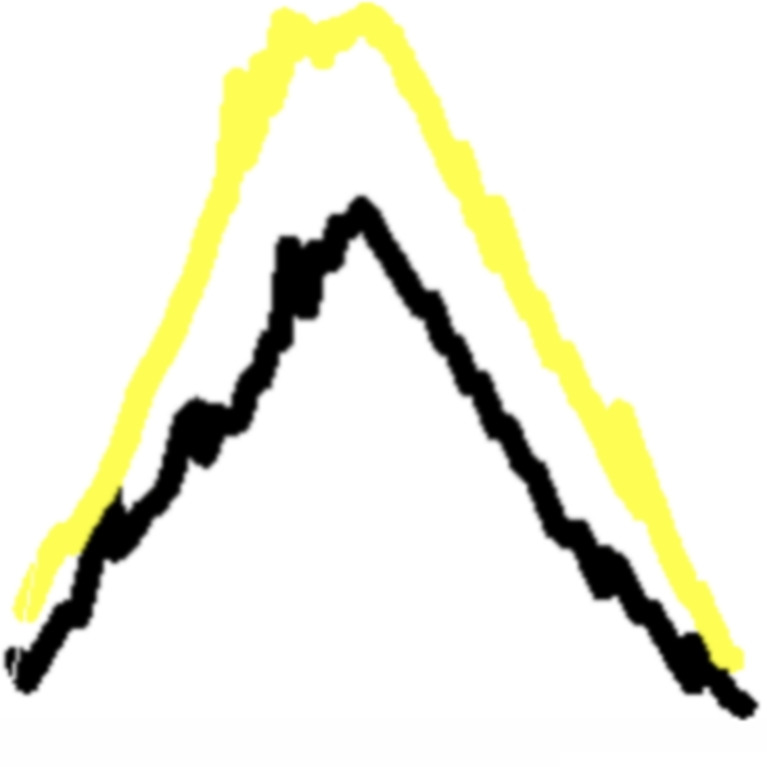
Fracture pattern extracted from a real humerus model and its two-dimensional representation for both cortical layers. The black line corresponds to the outer cortical surface and the yellow line to the inner cortical surface

In Fig. 16, the fracture pattern (Fig. 17) applied to the bone models can be observed. In the fracture pattern image, two distinct lines are depicted, one in black and the other in yellow.

One line represents the outer cortical surface, while the other corresponds to the inner surface. Despite sharing basic characteristics, discrepancies in their cut points arise due to variations in the fracture line as it traverses the cortical tissue.

**Fig. 18:**
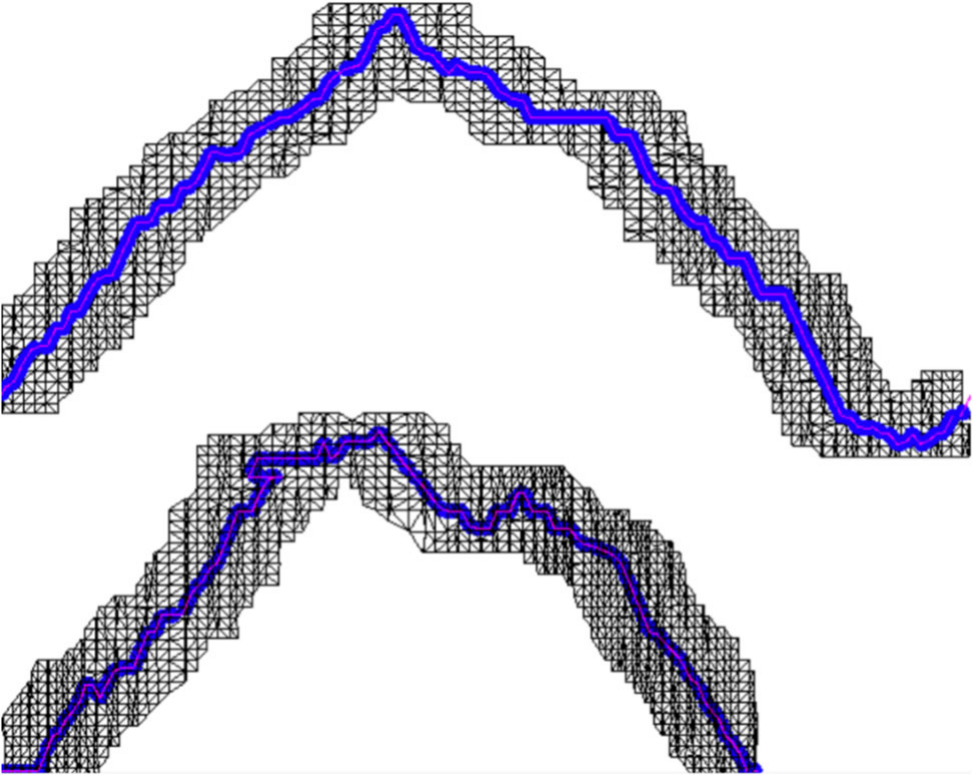
Two-dimensional representation of the fracture zone in both cortical layers of a femur model, simulating a real fibula fracture. Blue points indicate the vertices of the triangles selected for the subdivision

The projection of the fracture pattern onto the cortical tissue can be seen in Fig. 16. Figure 18 depicts the cutting points and the triangulation for this fracture.

### Height map generation

After obtaining the different bone fragments following the application of fracture patterns, height maps are generated for both the real (Fig. 11) and simulated models (Fig. 12 (6)). It is expected that the height maps will differ between the real and simulated models, as the pattern is applied to a different bone model in terms of size and geometry. The original pattern was derived from a fibula and humerus, tailored to the thickness of those models.

This process captures the 3D surface irregularities of the fracture and represents them as height values. By aligning the fracture zones of real and simulated models at the same location, differences in height can be assessed across the same reference region. These maps are then compared by computing average height differences and identifying deviations in surface profile, thereby validating how closely the simulation replicates the real fracture’s structure.

When applying the pattern to the new models (femur and humerus), it is scaled to fit the model’s thickness, defined by the bone’s generalized radius and resized along the *Y*-axis. This scaling process involves adjusting the results obtained during simulation to account for size differences between the original bone model and the target model receiving the fracture pattern. If the original model has thickness $T_{original}$ and the new model has thickness $T_{new}$, values from a height map or other measurements in the new model are scaled proportionally to compare properly with the values in the original model. The scaling is performed using the ratio $\frac{T_{original}}{T_{new}}$. This adjustment ensures that measurements in the new model align correctly relative to the original model, considering their dimensional disparities.

The arrangement of the height map coincides in both models at the fracture edge when making the cut. To verify that both contours correspond to the same fracture pattern, the average value of the height map for both the real and simulated cases was compared. The obtained results had to be resized due to the scaling of the fracture pattern during projection onto the model, owing to the difference in size between the original bone model and the model to which the fracture pattern is applied.

### Comparison of height maps

The validation process of our simulation models involves comparing the simulated fracture patterns with those obtained from CT scans of real fractured bones. This comparison serves as an indirect verification method. We will elaborate on this validation process and discuss future steps that include direct experimental verification through biomechanical testing.

**Table 4:** Quantitative comparison between simulated and real fracture surfaces sharing identical fracture patterns, based on height map metrics

Real model				Simulated model						
Model	Radius	Height map range	MMAR	Model	Radius	Height map range	MMAS	MMAS scaled	Distance between MMAR and MMAS scaled	Percentage variation of MMAR and MMAS scaled
Fibula	9.23	22.80	10.89	**Femur**	13.08	35.33	12.17	11.13	0.24	$-$2.22%
Fibula	9.23	22.80	10.89	**Humerus**	8.94	24.82	11.83	10.53	$-$0.36	3.32%
Humerus	5.00	24.00	13.00	**Femur**	13.08	271.41	62.41	14.44	1.44	$-$11.05%

*MMAR* (mean map from real fracture) and *MMAS* (mean map from simulated fracture) represent the average height values computed from the corresponding fracture surfaces. **Left:** Real model parameters, including bone type, radius, height map range (i.e., amplitude of height variation), and *MMAR* values. **Middle:** Simulated model parameters, including bone type, radius, height map range, *MMAS* values, and *MMAS* values scaled to match the real bone dimensions. **Right:** Direct comparison between real and simulated results, showing the difference between *MMAR* and scaled *MMAS*, as well as the percentage variation

Table 4 presents the data for both the real model and the simulated model. For each of the real models, it shows the generalized bone radius, the height or range of the height map (difference between the minimum and maximum points of the height map of the model), and the average distance of the height map in the fracture zone (*MMAR*). Following each real fractured bone model, the model onto which the fracture was simulated is presented, along with its generalized radius, the value of the height range in the fracture zone, the average distances of the height map, and the average distances of the height map properly resized (*MMAS*) or scaled for the simulated model (*MMAS* scaled). Finally, the difference between the average distances of the height map of the real fracture and the simulated model after resizing is shown as a percentage.

In these results (Table 4), we observe that the difference in the average distances in the height map between the real and simulated models shows very little variation, despite differences in the geometry and scale between the real and simulated bones (different source and destination bones). This variation ranges between 2 and 3% for the real fibula model and 11% for the real humerus model.

The latter value shows a higher variation due to the size disparity between the humerus and fibula, with radius of 5 and 13.08, and a height map range of 24.00 and 271.41 respectively. The number of triangles in the models also varies significantly, resulting in more cut points in the simulated model when applying fracture patterns, leading to a higher number of points in that model and, consequently, a variation in the average height map of the simulated model in areas with a higher density of cut points. Additionally, in the case of the humerus, the model had low resolution and a very small size, leading to a large difference in dimensions between the real model and the bone used for simulation. For this reason, the scale of the real bone (humerus) was adapted beforehand.

### Visual validation

Finally, the results obtained on the bone models were visually validated (Figs. 14 and 15) by a group of experts from the University Hospital of Jaén composed of two traumatologists, a radiologist and a surgeon specializing in fracture reduction, who confirmed that the simulated models accurately represented real fractures, similar to those observed in real bone models obtained through medical imaging. Some of their assessments determine that by comparing actual patterns that have been obtained from real fractures, the fragment generation is faithful to the fragmentation that would occur in a real fracture. Therefore, they determine that the fragment generation method aligns with the results that would be expected in a real fracture. They also point out that the inclusion of perturbations in the fracture zone makes it more similar to the appearance of a real fracture as the union between the two layers of cortical tissue is not so clearly perceived.

## Discussion

The experiments in this study emphasize the significance of bone fracture simulations, addressing the challenges of real-time fracture observation and analysis. Fractures occur unpredictably, making it impossible to observe their progression or predict the exact moment of injury under natural conditions.

Laboratory replication also faces significant hurdles. Variables such as the presence or absence of adjacent tissues and whether the subject is alive differ markedly from natural fracture scenarios, preventing accurate reproduction of fracture events in controlled experimental settings.

The bone models developed here offer a practical tool for studying fractures. By using medical imaging data and patterns validated through forensic analysis, these models replicate fracture patterns with high accuracy, providing insights into bone behavior that are difficult to observe directly in clinical or experimental practice.

Fracture generation starts with selecting and adapting a fracture pattern to a 3D bone model, ensuring both internal and external cortical layers are represented. Subdivision of the model using Delaunay triangulation and the application of perturbation techniques generate irregularities that enhance realism, mimicking the surface non-uniformity typically observed in real fractures. However, localized defects or impressions, such as those caused by bone crushing or impact, are not explicitly simulated. Future work may include the integration of defect-specific deformation models to better represent high-impact or osteoporotic fracture scenarios.

To ensure precise fracture control, a method generates two-dimensional patterns projected onto three-dimensional bone models. This approach accurately represents fracture characteristics and incorporates automatic forensic validation, enhancing pattern authenticity and reliability. An additional validation method uses real fractured bone models from CT scans applied to intact bones. Though requiring careful adaptation, this technique effectively replicates specific patient-observed fractures.

The simulation methodology supports complex fracture geometries, including branching and the generation of multiple fragments, especially when fracture patterns are manually designed or generated parametrically. In such cases, the algorithm evaluates mesh continuity and separates affected regions into distinct fragments when topological disconnection occurs. This allows the simulation of comminuted and ramified fractures. However, when the simulation is based on a real CT scan, only the available scanned fragments are represented. Due to scan limitations, clinical positioning, or partial reconstruction, the full fracture context is often not captured, restricting the simulation to the geometry of a single isolated fragment. This represents a practical limitation when simulating complex clinical fractures directly from CT data.

Compared to existing methods, our approach balances anatomical realism with practical applicability. FEA and physics-based models, although useful for biomechanical insights, require high-resolution CT scans, precise material properties, and complex calibration procedures, factors that limit their scalability and integration into standard clinical workflows [10]. Similarly, machine learning-based approaches depend on large annotated datasets and often lack interpretability, making them less adaptable to rare or complex fracture scenarios.

In contrast, the proposed methodology generates geometrically realistic fracture models using validated 2D fracture patterns, either manually designed, parametrically generated, or extracted from real clinical cases, without requiring individualized CT imaging. This reduces reliance on medical imaging resources, simplifies preprocessing requirements, and enables the rapid generation of diverse fracture configurations.

Furthermore, the method offers fine control over fracture morphology, propagation trajectory and surface complexity through adjustable pattern parameters and perturbation techniques. Its modular and reproducible nature makes it well-suited for a wide range of applications, including surgical training, preoperative planning, forensic analysis, biomechanical simulations, synthetic dataset generation, training environments based on virtual reality, and physical model fabrication via 3D printing. Compatibility with standard mesh formats and simulation workflows further enhances its versatility across clinical, educational, and research domains.

## Conclusions and future work

Our study presents a novel approach to simulating bone fractures using two-dimensional and three-dimensional models. We investigated two methodologies: generating fractures from two-dimensional patterns and applying real fracture patterns to bone models. Both methods simulated fractures effectively and captured their distinctive characteristics. Minimal variation between real and simulated fractures demonstrates the robustness of these methods. Specifically, the distance between MMAR and MMAS scaled values varies between $-$0.36 and 1.44, while the percentage variation ranges from $-$11.05 to 3.32% (see Table 4), confirming the accuracy of the simulation.

The resulting models constitute a versatile dataset for use in research, surgical training, planning, and education. This work establishes a solid foundation for advancing fracture simulation, offering valuable tools for medical and healthcare research.

While the methodology is designed to be generalizable to different anatomical regions, certain components of the pipeline may require specific adaptations. In particular, the projection strategy used for long bones relies on their elongated morphology and well-defined anatomical axes. These assumptions may not hold in irregular bones or articular surfaces, where alternative projection and transformation methods may be required. By contrast, other stages of the process, such as extracting fracture zones from CT scans and transferring 2D patterns onto 3D models, can still be applied with minimal modification. Addressing these anatomical and geometric variations will be an important direction for future development.

Future directions include refining simulation techniques to enhance accuracy through advanced computational methods and machine learning. Clinical validation studies involving healthcare professionals and patient data will assess the real-world applicability of simulated fractures. Incorporating trabecular bone into models and simulating real geometries from CT scans will broaden the research scope.

Validation will expand to include biomechanical analyses, such as stress distribution and fracture propagation patterns. Integrating simulated fractures into surgical training, biomechanics, education, and imaging technologies can improve fracture management strategies. Exploring applications in orthopedic research, biomedical engineering, and forensic science will further enhance their utility, contributing to advancements in bone fracture understanding and care.
